# A case of ectopic pancreas in the ileum presenting as obscure gastrointestinal bleeding and abdominal pain

**DOI:** 10.1186/s12876-019-0971-7

**Published:** 2019-04-17

**Authors:** Rugile Mickuniene, Ieva Stundiene, Tomas Jucaitis, Dileta Valanciene, Jonas Valantinas

**Affiliations:** 1 Clinic of Gastroenterology, Nephrourology and Surgery, Vilnius University, Santariskiu street 2, 08406 Vilnius, LT Lithuania; 2 Department of Radiology, Nuclear Medicine and Physics of Medicine, Centre for Radiology and Nuclear Medicine, Vilnius University, Vilnius, Lithuania

**Keywords:** Ectopic pancreas, Ileum, Gastrointestinal bleeding

## Abstract

**Background:**

Ectopic pancreas is an infrequent submucosal tumor in the gastrointestinal tract defined as a pancreatic tissue lacking vascular or anatomic continuity with the main body of the pancreas. Ectopic pancreas in the ileum is a rare and often an incidental finding. We report a case of ectopic pancreas in the ileum causing obscure gastrointestinal bleeding and episodes of abdominal pain.

**Case presentation:**

59-year-old man with 3 months history of intermittent melena, accompanied by the episodes of abdominal pain in the left upper quadrant and generalized fatigue was admitted to our department. The investigations showed that the patient had a low hemoglobin level, i.e. 10.9 g/dL with hypochromic microcytic anemia pattern seen in complete blood count (MCV 70.2 fl, MCH 21.4 pg). Esophagogastroduodenoscopy and colonoscopy did not reveal any abnormalities. Magnetic resonance enterography revealed a large (2.5 × 2.3 cm) pedunculated polyp in the ileum. Examination by single-balloon enteroscopy revealed a polyp with long pedicle located approximately 1.5 m distal to terminal ileum. Polypectomy was performed. Histopathologic examination stated, that the specimen contained ectopic pancreatic tissue which was involving muscular layer of the ileum. Ectopic pancreatic tissue included acinar cells and cystically dilated secretory ducts without islets of Langerhans.

**Conclusion:**

Our case report reveals a very rare cause of obscure gastrointestinal bleeding accompanied by the episodes of abdominal pain – an ectopic pancreas located in the ileum.

## Background

Ectopic pancreas (also referred as heterotopic pancreas, pancreatic heterotopia, accessory pancreas, aberrant pancreas, or pancreatic rest) is an infrequent submucosal tumor in the gastrointestinal (GI) tract, defined as a pancreatic tissue lacking vascular or anatomic continuity with the main body of the pancreas [1–3]. The incidence varies between 0.5–13.7% on autopsy studies, with 70–90% of the lesions discovered in the stomach, duodenum or jejunum [1, 2, 4, 5]. Ectopic pancreas in the ileum is a rare and often an incidental finding. The incidence is about 3.8% [6]. Ectopic pancreas is usually a silent anomaly, but it may become symptomatic when complicated by pathologic changes such as inflammation, bleeding, obstruction, or malignant transformation [5, 7, 8]. We report a case of ectopic pancreas in the ileum causing recurrent obscure GI bleeding and episodes of abdominal pain.

### Case presentation

A 59-year-old man with 3 months history of intermittent melena accompanied by the episodes of abdominal pain in the left upper quadrant and generalized fatigue was admitted to the department. He denied any other change in bowel habits or a history of hemorrhoids and was referred to hospital for evaluation of the GI bleeding. Patient’s medical history did not include any previous diagnoses. He was not taking any medications. At the time he was a non-smoker and did not consume any alcoholic drinks or recreational drugs. The patient also had two repeated episodes of left upper quadrant abdominal pain and dark black tarry feces within the last 3 months prior to admission to our hospital. However, symptoms resolved spontaneously and the patient did not make an appointment to see the doctor.

On examination the patient was pale, the abdomen was tender in the left upper abdominal area with no signs of rebound tenderness, no lump was palpable. Digital rectal examination revealed melena; the rest of the examination was unremarkable. The investigations showed that the patient had a low hemoglobin level, i.e. 10.9 g/dL with hypochromic microcytic anemia pattern seen in complete blood count (MCV 70.2 fl, MCH 21.4 pg). In addition, the patient had low serum iron, i.e. 6.4 μmol/L (normal range 9.5–29.9 μmol/L) and low ferritin levels, i.e. 28.8 μg/L (normal range 20–300 μg/L). The carcinoembryonic antigen level was 1.2 μg/L (normal < 5.00 μg/L). Other routine blood tests including lipase, plain chest and abdominal X rays along with abdominal ultrasound, esophagogastroduodenoscopy and colonoscopy were unremarkable. Thorough conventional evaluation of GI bleeding has failed to reveal a source, therefore, it was rational to proceed with further investigation of the small intestine.

Usually most cases of bleeding in the small intestine are caused by abnormal blood vessels in the wall of bowel - angioectasias, angiodysplasias, or arteriovenous malformations. However, there are many other possible causes of bleeding in the small intestine, including Crohn’s disease, benign and malignant tumors, polyps and ulcers.

**Fig. 1:**
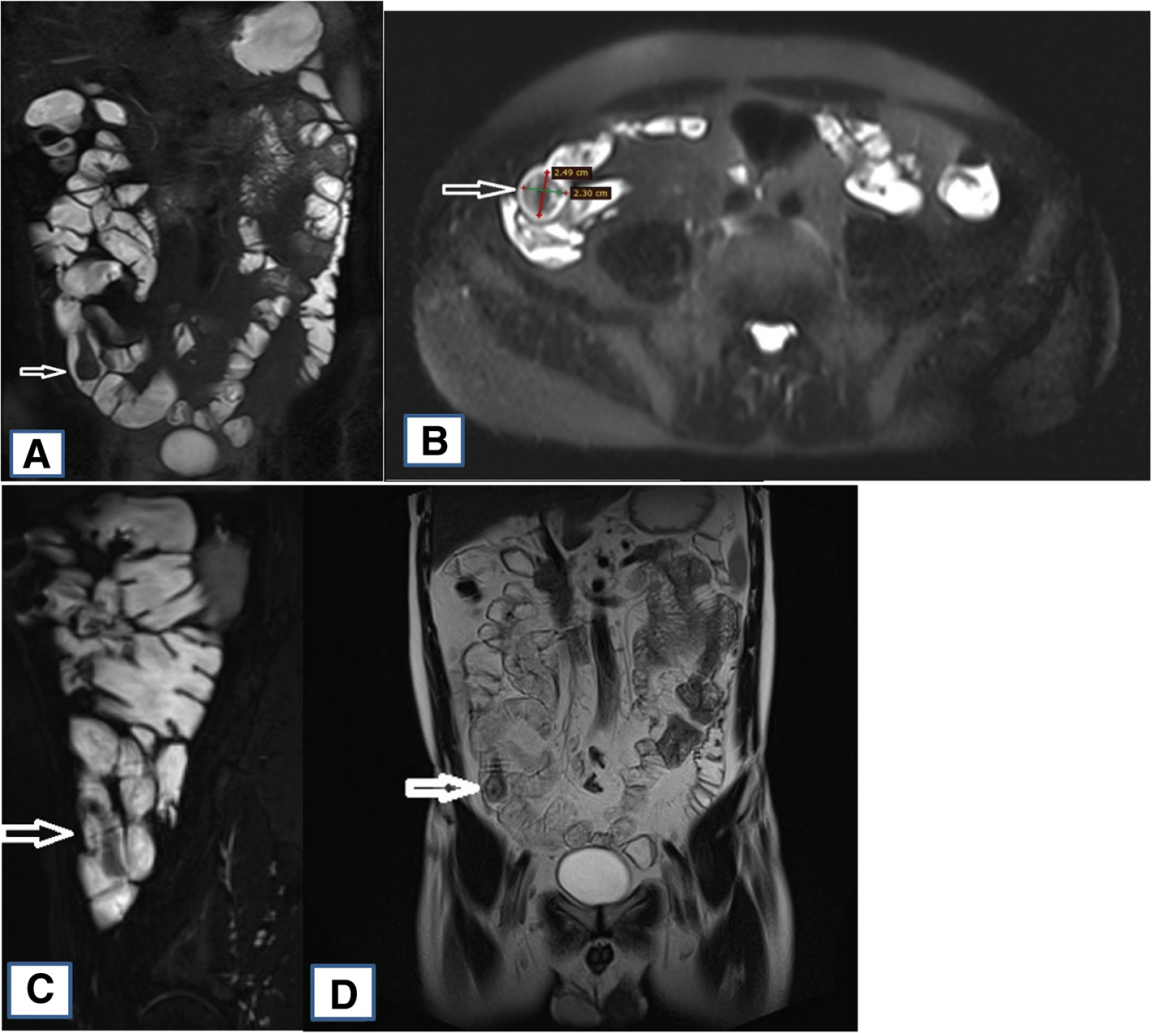
MR enterography: large pedunculated polyp. **a** Coronal T2-weighted image, mannitol as a luminal contrast, no intravenous contrast. **b** Axial T2-weighted image, mannitol as a luminal contrast, no intravenous contrast (polyp size 2.5 × 2.3 cm). **c** Sagittal T2-weighted blade fat saturation (fs) image, mannitol as a luminal contrast, no intravenous contrast (good view of long pedunculus). **d** Coronal T1-weighted blade image, mannitol as a luminal contrast, no intravenous contrast. **e** Coronal T1-weighted vibe fs image, intravenous contrast

Unfortunately, the capsule endoscopy is not reimbursed by Patient Sickness Fund in Lithuania, therefore we performed magnetic resonance (MR) enterography to help visualise possible bleeding site in the small bowel. MR enterography revealed a large pedunculated (attached to the intestinal wall by a 3 cm length pedicle) polyp, measuring approximately 2.5 × 2.3 cm and involving middle third of the ileum **(**Fig. 1.**)**. Furthermore, ulceration marks at the top of the polyp were described.

**Fig. 2:**
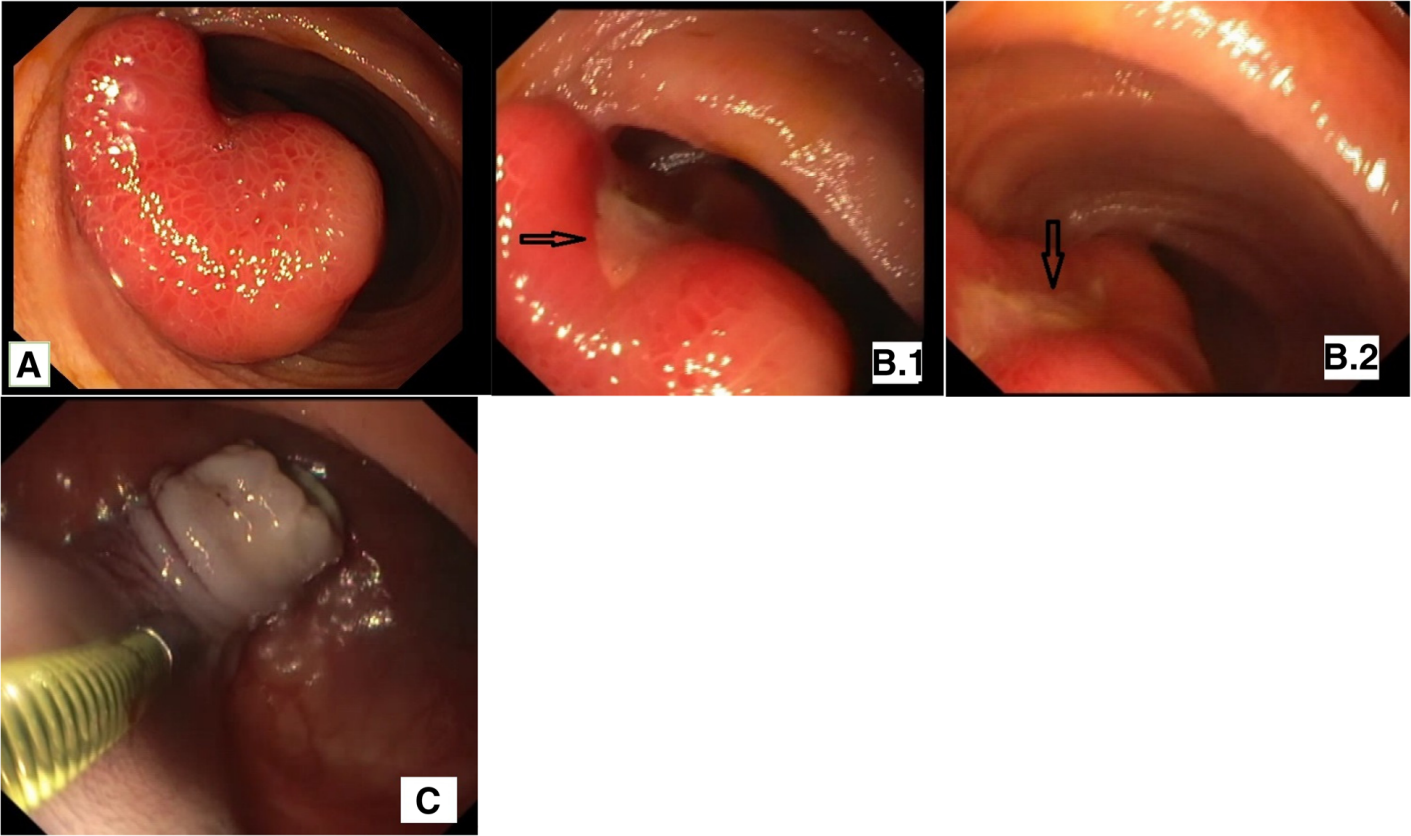
Single-balloon Enteroscopy (SBE): **a** Pedunculated polyp located in the ileum. **b**.**1–2** Ulceration of the polyp. **c** Remnant of the pedicle after polypectomy and dilution of adrenaline injection

For further investigation, the patient underwent retrograde single-balloon enteroscopy (SBE) to directly visualize pedunculated polyp, described previously on MR enterography. Examination by SBE revealed a polyp with a long pedicle located approximately 1.5 m distal to the terminal ileum **(**Fig. 2.**)**. Endoloop-Assisted polypectomy was performed. However, the procedure was complicated with postpolypectomy bleeding from the pedicle. Dilution of adrenaline 20 ml (1/10.000) was injected into the bleeding area and the bleeding was controlled.

**Fig. 3:**
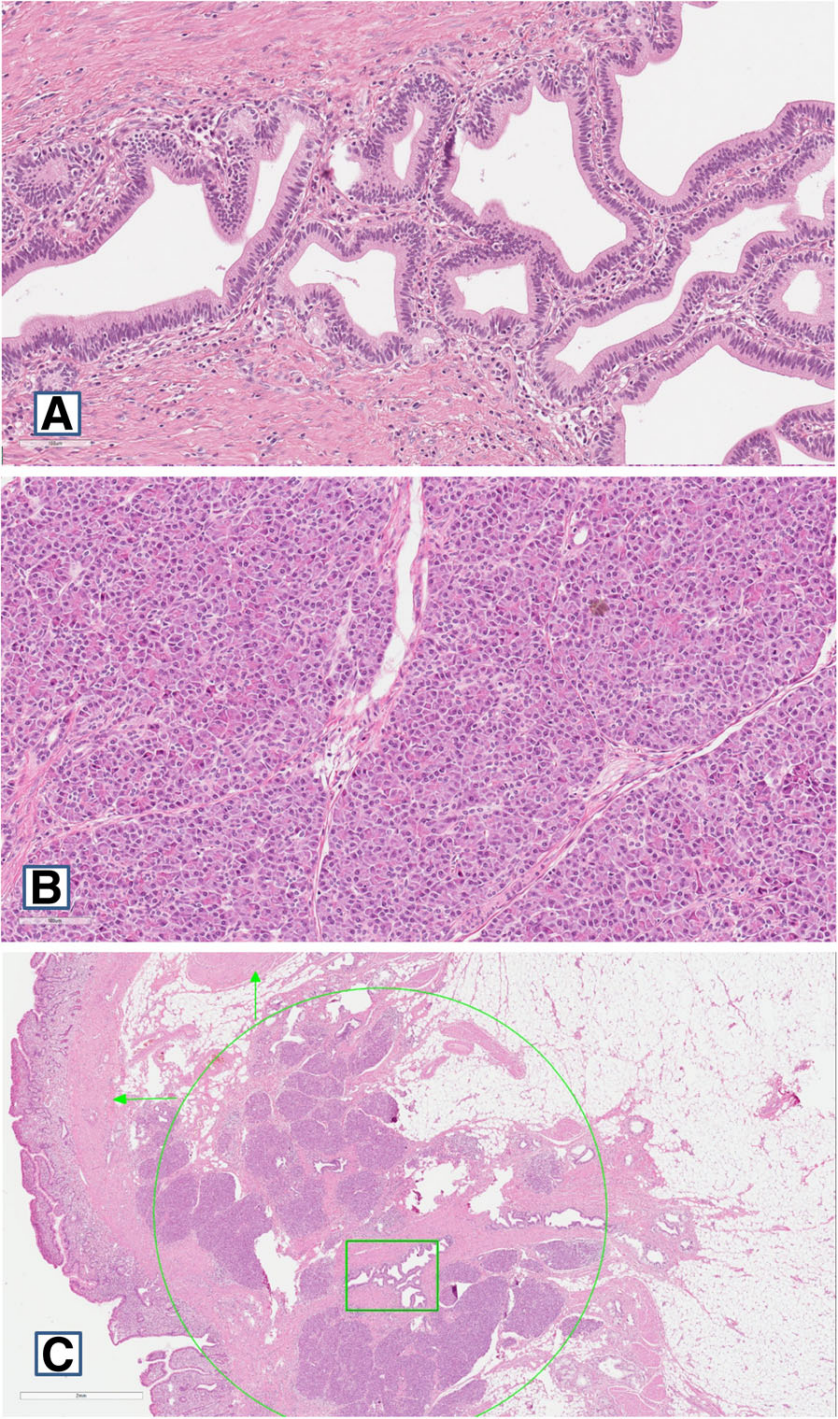
Histology of ectopic pancreatic tissue: **a** Dilated ducts of ectopic pancreas **b** Acini of ectopic pancreas **c** Whole ectopic pancreatic tissue (green circle), dilated ducts (green rectangle), longitudinal muscle layer (vertical green arrow), circular muscle layer (horizontal green arrow)

Brownish polyp with rugged surface was noted in the gross specimen. Cross-section of the polyp revealed a yellow node sized 1.5x1x1 cm. In addition, histopathological examination was performed. The report stated that the specimen contained ectopic pancreatic tissue involving longitudinal muscle layer of the ileum (Fig. 3.**)**. Ectopic pancreatic tissue included acinar cells and cystically dilated secretory ducts without islets of Langerhans. Also, there was evidence of mucosal ulceration of the ileum.

The patient recovered after the enteroscopy well and had no further GI symptoms (since discharge until the time of writing).

## Discussion and Conclusions

Ectopic pancreas is a congenital anomaly, in which pancreatic tissues lacking anatomical or vascular connections with the normal pancreas may be found anywhere within the abdominal cavity other than its usual location [3]. Ectopic pancreas was first reported by Jean-Schultz in 1727 [4, 6, 8].

Ectopic pancreas can be found throughout the entire gastrointestinal tract, however, studies indicate that 70–90% of cases are located in the upper gut, including the stomach (25–47%), duodenum (11.7–36.3%) and jejunum (15–35%) [1, 2, 5, 6]. Ectopic pancreas found in the ileum is a rare entity (3.8%) [5–7].

According to literature, ectopic pancreas is mainly located in submucosa (54–75% of cases, also, it may span the submucosa and muscularis propria in 23% of cases), following by muscular layer (muscularis propria in 8% of cases) and serous layer (11–13% of cases). Lesions which are located in the stomach and duodenal bulb may involve full thickness of the wall (4% of cases) [4, 9, 10]. In the presented case the ectopic pancreatic tissue was located in the longitudinal muscle layer of the ileum.

Although the pathogenesis of ectopic pancreas remains unknown, based on literature there are two main theories that have been proposed to explain occurrence of ectopic pancreas - misplacement theory and metaplasia theory. The most widely held misplacement theory claims that during the period of embryonic rotation of the dorsal and ventral buds deposits of pancreatic tissue migrate from the main body of pancreas and are implanted at various ectopic sites [2, 5, 7, 8]. On the other hand, metaplasia theory implicates that during embryogenesis endodermal tissues migrate to the submucosa and then turn into pancreatic tissue [4]. As we already mentioned before ectopic pancreas is usually an incidental finding and typically asymptomatic, but it may become clinically evident when complicated by inflammation, bleeding, obstruction or malignant transformation [5, 7, 8]. Symptoms depend on the site where ectopic pancreas is located. Since it is usually found in the upper GI, dyspepsia, epigastric pain, nausea, vomiting, jaundice, abdominal fullness, anorexia, weight loss, anemia and hematemesis are the most common presentation. In addition, there is a possibility of GI obstruction due to the mass effect caused by ectopic pancreas [1, 11, 12]. The most common presentation of the small intestine is obstruction or intussusception [1]. Ectopic pancreas of the ileum, on the other hand, is very rare, usually has no symptoms and is found incidentally during the surgery for other reasons, and very rarely as a leading point for intussusception [7].

Ectopic tissue is capable of reproducing same pathologic conditions that can affect the orthotopic pancreas, therefore pancreatitis, formation of cysts and pseudocysts, neuroendocrine syndromes, benign and malignant neoplasms can occur [1, 4, 13]. Cyst and pseudocyst formation results from the exocrine duct obstruction (retention cysts) or secondary to pancreatitis [1, 4]. The incidence of malignant transformation of ectopic pancreas is very low, occurring only in 0.7–1.8% of cases. The most common malignancy is ductal adenocarcinoma. Other malignant tumors that have been reported are mucinous cystadenocarcinoma, acinar cell carcinoma, islet cell tumor, and solid and papillary neoplasms [4]. Malignant transformation is therefore a differential diagnosis and should be excluded [13].

It has been noticed, that severity of symptoms depends on ectopic lesion size, hence lesions larger than 1.5 cm were associated with more severe symptoms [1, 8, 14]. Abdominal pain is one of the most common symptoms. The most reasonable explanation is that the pain is caused by secretion of hormones and enzymes triggering the onset of spasms, chemical irritation and inflammation of surrounding tissue [1, 8, 14]. Histopathological evidence of chronic inflammation is described as peri-ductal or intra-lobular fibrosis and ductal dilatation [1]. The latter was seen in our histopathological specimen of ectopic pancreas. This type of histopathological lesions may be caused by repeated episodes of inflammation of pancreas (acute pancreatitis), same way as it appears in normal pancreas. Clinically our patient complained of intermittent left upper quadrant abdominal pain, meanwhile ectopic pancreas was in the right lower part on MR examination. In our opinion, abdominal pain might be explained by the episodes of acute pancreatitis, active exocrine function of ectopic pancreas located in the ileum. It may also be the result of hemorrhage in the lesion due to mucosal erosion. Active pancreatic enzymes, such as amylase and trypsin might flow through the intestinal lumen and produce inflammation and spasm not only locally, but also distant from the actual ectopic mass site. Ileum is a mobile intestine with an active peristalsis and its position in the abdominal cavity varies greatly. The polyp in our case was quite large (~ 3 cm) with long pedicle (~ 3 cm), therefore pain localization could have been different. An intermittent mechanical obstruction (sub-ileus) of the intestinal lumen or recurrent itussusception could have also been caused by the polyp which was quite large with a long pedicle or due to tissue edema. Muscular and submucosal wall proximity of the pancreatic tissue is believed to cause bowel dysmotility and act as local disturbance leading to intussusception [15]. Intussusception due to ectopic pancreas in ileum has been described in a few cases, including pediatric as well as adult patients [15–17]. Furthermore, ectopic pancreas in the small bowel may be a rare cause of obscure GI bleeding [5]. Because this morbidity is very rare, the prevalence of GI bleeding due to ectopic pancreas is not well known. According to literature, GI bleeding has been reported in 3 out of 73 symptomatic cases among 212 patients with ectopic pancreas diagnosis [18] and in 5 out of 15 symptomatic cases among 39 patients with ectopic pancreas diagnosis [19]. Obscure GI bleeding occupies about 5% of all GI bleedings and almost 75% of these are thought to be localized in the small intestine [20]. GI bleeding presenting with melena due to mucosal erosion, ulcer formation, especially if ectopic pancreas is located in the small intestine, has been reported [21]. Our case report consists of episodes of obscure bleeding from ileal polyp, as patient presented with anemia, intermittent melena and the ulceration of the polyp was confirmed by MR enterography, enteroscopy and histopathological examination.

Ectopic pancreas has several characteristic radiographic and endoscopic features that may lead to its identification. During esophagogastroduodenoscopy the ectopic pancreas classically presents as soft rubbery well-circumscribed broad based yellow submucosal lesion (ranging from 1 mm to 5 cm) [1, 2, 8, 11, 14, 22]. The characteristic central umbilication represents the orifice of the ductal system [12, 22]. Though, lesions of ectopic pancreas which are smaller than 1.5 cm usually do not show such an orifice [1]. The characteristic feature of umbilication is reported in less than half of the cases, therefore ectopic pancreas may resemble other submucosal tumors during the endoscopic examination [8]. Radiological studies, such as barium swallow series may show nonspecific fold thickening with rounded filling defects and sometimes a typical central indentation, which helps to differentiate it from other types of intramural masses [1, 2, 4, 8, 11]. On the other hand, all the described features are not always present leading to a difficulty in diagnosis [12]. It is important to emphasize, that various radiologic tests are almost always performed to search for more common causes of abdominal symptoms, and ectopic pancreas is usually an incidental finding. Computed tomographic findings are usually nonspecific, although in examination with intravenous contrast ectopic pancreatic tissue can enhance to the same degree as orthotopic pancreas. Also, computed tomography may show exophytic bowel wall lesions or mural wall thickening, luminal or compressive obstructions [12]. The magnetic resonance imaging appearance of ectopic pancreas is similar to the normal pancreas [4]. Capsule endoscopy and enteroscopy are being used to find small bowel lesions which were previously inaccessible without surgery. The main disadvantage of capsule endoscopy is that biopsy from the lesion is not allowed. On the other hand, enteroscopy allows to perform proper visualization and diagnostic/therapeutic procedures in all segments of the small intestine [2]. Even if ectopic pancreas lesion is identified endoscopically biopsies are often too superficial and nondiagnostic, because this lesion is submucosal [4, 8, 11, 12, 23]. Endoscopic ultrasound is the golden standard to evaluate submucosal lesions of the GI tract. Characteristic features of ectopic pancreas are indistinct margins, heterogeneous, hypoechoic appearance, and location within either the third and fourth layers (fusion type) or only in the third layer (separate type) [1, 9]. However, none of these findings are diagnostic of ectopic pancreas. Unfortunately, aspirated biopsy specimen taken during endoscopic ultrasound is often nondiagnostic and the cost of the procedure is high, therefore, surgery is often considered [19].

Diagnosis of ectopic pancreas is easier in the upper GI tract, where it may present characteristic features. On the other hand, the distal small bowel is notoriously difficult to visualize, therefore diagnosis of ectopic pancreas in the ileum is a rare and usually incidental.

Imaging studies and endoscopic visualization are neither specific nor sensitive enough to make diagnosis of ectopic pancreas and histopathological examination remains the gold standard to confirm this anomaly. In our case report fusion of clinical and radiological evaluation finally led us to endoscopic resection of the polyp and a definitive histopathological diagnosis of ectopic pancreas.

The first case of ectopic pancreas in ileum presented as gastrointestinal bleeding was reported by Tanigawa et al. in 1993 [24]. In this case report they highlighted the diagnostic difficulty to make such diagnosis and showed the possibility of diagnosing rare abnormalities of the ileum.

In another case reported by Huan-Lin Chen et al., authors also describe ectopic pancreas in ileum presented as GI bleeding, which was visualised by capsule endoscopy. Among patients with obscure GI bleeding capsule endoscopy has a high sensitivity and specificity for bleeding source detection and is well-suited as the first choice for evaluating obscure GI bleeding [23]. As mentioned before, capsule endoscopy is not reimbursed by the Patient Sickness Fund on in Lithuania, therefore, we performed magnetic resonance (MR) enterography to help visualise possible bleeding site in the small bowel. Other diagnostic options include enteroscopy, barium contrast small bowel studies and angiography. In Huan-Lin Chen et al. case, laparotomy was performed to look for and to remove the ileal polyp. Until recently the only therapeutic option to remove distal small intestine lesions and to receive histology of the tissue in order to make definite diagnosis was surgical resection [22]. With progression in deep small bowel enteroscopy biopsy and endoscopic resection of small bowel lesions are becoming more ordinary. As we described in our case report, endoscopic resection may be a rational, sufficiently safe and less invasive option for removal of some small bowel polypoid lesions.

Mi-Jeong Lee at al., Qun-Ying Wang et al. and Woo Hyung Choi et al. all described cases in which ectopic pancreas was found in jejunum and presented as gastrointestinal bleeding (melena ± hematochezia or stubborn anemia with positive fecal occult blood test). In all three reports lesions were found by capsule endoscopy and in two case reports patient’s examination was continued by barium small bowel series to find the exact location. Finally, patients underwent surgery (laparoscopy or laparotomy) to remove lesion, recovered well and symptoms never reoccurred. In the end, histological examination revealed submucosal ectopic pancreatic tissues [9, 19, 23].

For symptomatic ectopic pancreas patients local surgical or endoscopical resection of the lesion seems to be the most appropriate therapy [9, 13]. Which type of the procedure should be performed depends on the type and size of the ectopic pancreatic tissue. Ectopic pancreas originated from the mucosa or submucosa can be removed with mucosal resection of transparent cap during endoscopy [9]. However, according to authors, surgery remains the standard therapy [13]. Furthermore, lesion should be removed to prevent complications and the need for reoperation when it is found incidentally during surgery [1, 8, 13] and when it is larger than 3 cm in size [8]. Also, authors emphasize that more common causes of abdominal complaints such as peptic ulcer disease, gastro-esophageal reflux disease and biliary disease have to be ruled out before any surgical procedure [12]. When ectopic pancreas is associated with bleeding, GI obstruction or suspicion of malignant transformation the appropriate surgical intervention is imposed [1]. If malignant transformation is suspected extended oncological surgery is justified [13]. Asymptomatic patients with histologically proven diagnosis and when malignancy is definitely excluded must remain under medical supervision and have regular follow-ups [1, 9, 13]. However, intervals of the periodical surveillance are unknown. On the other hand, other authors suggest that there is no need for increased surveillance [12]. Ormarsson et al. [25] 13 years followed 32 patients with ectopic pancreas diagnosis of the stomach or small intestine and found no malignant transformation in any of the patients over this time.

Ectopic pancreas was classified by Heinrich in 1909. Type I is composed of acini, ducts and endocrine islet cells, Type II includes acini and ducts without islet cells, and Type III includes only pancreatic ducts [4, 13]. Most common histopathological presentation features all three components of the normal pancreas (Type I) [4, 13], however, in our case report ectopic pancreas included acini and ducts without islet cells (Type II). Gaspar Fuentes et al. in 1973 further modified this classification and included fourth type of ectopic pancreas, which contained only islet cells [2, 4]. The definitive diagnosis of heterotopic pancreas is made by histopathological examination of the tissue [8].

Our case report reveals a very rare cause of obscure gastrointestinal bleeding accompanied with episodes of abdominal pain – an ectopic pancreas located in the ileum. Although ileal polyp was located by MR enterography and later enteroscopy, diagnosis of ectopic pancreas could not be made until polypectomy and histopathological examination.

## Abbreviations

GI: Gastrointestinal
MR: Magnetic resonance
SBE: Single-balloon enteroscopy
